# Brugada Pattern Electrocardiogram Unmasked with Cocaine Ingestion

**DOI:** 10.1155/2013/704859

**Published:** 2013-02-17

**Authors:** M. Chadi Alraies, Mohammed A. R. Chamsi-Pasha, Motaz Baibars, Abdul Hamid Alraiyes, Khaldoon Shaheen

**Affiliations:** ^1^Department of Hospital Medicine, Cleveland Clinic Lerner College of Medicine of Case Western Reserve University, 9500 Euclid Avenue, Mail Code A13, Cleveland, OH 44195, USA; ^2^Department of Internal Medicine, Cleveland Clinic, Cleveland, OH 44195, USA; ^3^Department of Hospital Medicine, Peninsula Regional Medical Center, Salisbury, MD 70118, USA; ^4^Tulane University Health Sciences Center, Pulmonary Diseases, Critical Care and Environmental Medicine, New Orleans, LA, USA

## Abstract

Cocaine is considered a leading cause of drug-related deaths. This is usually sudden, unwitnessed, and without prodromal features. It has been reported that in-hospital mortality is close to 2%. Cocaine has powerful central nervous system effects^1^ and acute cocaine overdose has been associated with hyperthermia, agitation, paranoid ideation, status epilepticus, ventricular fibrillation, ventricular tachycardia, and myocardial infarction (MI). The mechanisms of cocaine-related death remain poorly understood. We report a patient who survived massive cocaine ingestion with psychomotor agitation and generalized seizures followed by asystolic cardiac arrest and transient Brugada pattern on electrocardiogram (ECG).

## 1. Introduction

The use of cocaine in the United States is increasing and the incidence of hospitalizations and deaths from cocaine overdose is escalating. The mechanisms of cocaine-related death remain poorly understood. Cocaine has been associated with different types of cardiac dysrhythmias, most commonly: supraventricular tachycardia, complete bundle-branch block, complete heart block, ventricular tachycardia, torsade de pointes, ventricular fibrillation, asystole, and Brugada pattern. We report a patient who survived massive cocaine ingestion with asystolic cardiac arrest and transient Brugada pattern on electrocardiogram (ECG).

## 2. The Case

**Figure 1:**
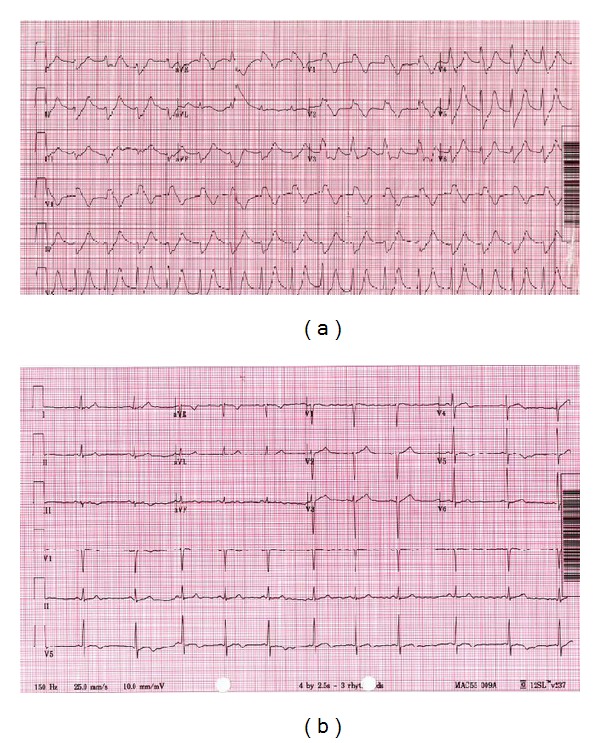
(a) Repeated ECG 10 minutes later showing accelerated idioventricular rhythm at 66 beats/min, QRS (0.44 sec), QTc (0.58 sec) with right bundle branch block (RBBB) and left anterior hemiblock (LAHB) configuration, and rSR' pattern with coved ST-segment elevation in V1 and V2 mimicking Brugada syndrome. (b) Normal sinus rhythm at 66 beats/min.

A previously healthy 27-year-old man ingested a bag of cocaine as an impulsive gesture to avoid police detection. One hour later, he developed psychomotor agitation followed by generalized tonic-clonic seizures. The patient was transported to the emergency department and the seizures subsequently terminated with intravenous lorazepam. Later, the patient was noticed to be in ventricular asystole. The patient was resuscitated to a spontaneous rhythm through which epinephrine and sodium bicarbonate were given. Following resuscitation, the patient was intubated and mechanically ventilated. On exam, temperature was 39°C, pulse 70/min, blood pressure 90/70 mmHg, respiratory rate 32/min, oxygen saturation 98% with full ventilator support (FiO_2_ 100%), and Glasgow Coma scale of 5/15. Chest examination revealed bibasilar crackles. Heart exam was significant for regular rate and rhythm with frequent escaped beats. Blood work was within normal limit except for bicarbonate of 5.4 mEq/L, anion gap of 36, and creatinine of 1.8 mg/dL. Arterial blood gas showed pH of 6.1, PCO_2_ of 86 mmHg, HCO_3_ of 5.4 mEq/L, and PO_2_ of 350 mmHg. Troponin I was 50.90 ng/mL. Urine toxicology screen was positive for cocaine. Electrocardiogram (ECG) (Figure 1(a)) showed accelerated idioventricular rhythm at 66 beats/min, prolonged QRS (0.440 sec), prolonged corrected QT (QTc) (0.533 sec) with right bundle branch block and left anterior hemiblock configuration. An rSR' pattern with coved ST-segment elevation was noted in V1 and V2 consistent with Brugada pattern. The patient was treated with sodium bicarbonate and lorazepam infusions. He quickly achieved hemodynamic stabilization with near normalization of serum bicarbonate and pH levels. Cardiac catheterization was done and showed normal coronaries. Few hours later, repeated ECG showed substantial QRS interval shortening (0.09 sec), resolution of ST-segment elevation with restoration of sinus rhythm (Figure 1(b)). Following 7 days in the hospital, the patient was discharged back to jail without complications.

## 3. Discussion

The use of cocaine in the United States is increasing and the incidence of hospitalizations and deaths from cocaine overdose is escalating [1, 2]. The prime mode of cocaine-induced death appears to be respiratory paralysis secondary to the effect of cocaine on the medullary portion of the brain [1]. In our case, this might explain the asystolic cardiac arrest because ventilation with supplemental oxygen was sufficient to restore spontaneous cardiac rhythm. Cocaine cardiovascular effects include ischemia—with or without infarction—and dysrhythmia [3]. The cardiac dysrhythmias ascribed to cocaine are supraventricular tachycardia, sinus bradycardia, accelerated idioventricular rhythm, complete bundle-branch block, complete heart block, ventricular tachycardia, torsade de pointes, ventricular fibrillation, asystole, and Brugada pattern which is defined as right bundle-branch block with ST-segment elevation in leads V1, V2, and V3 [1, 4]. Brugada phenotype is more prevalent in males [5]. Cocaine may affect the generation and conduction of cardiac impulses by several mechanisms [1]. First, as a sympathetic agonist, it may increase ventricular irritability and lower the threshold for fibrillation. Second, it inhibits the generation and conduction of the action potential (i.e., it prolongs the durations of the QRS and QT intervals) as a result of its sodium-channel-blocking effects [1, 6]. Thus, cocaine acts in a manner similar to that of a Class IA antiarrhythmic agent. Third, cocaine increases the intracellular calcium concentration, which may result in afterdepolarization and triggered ventricular arrhythmias. Fourth, it reduces vagal activity that potentiates cocaine's sympathomimetic effects. 

One of the unique features in our case is the transient Brugada pattern observed. Since its original description in 1992,^2^ the Brugada syndrome has created considerable interest among cardiac electrophysiologists. The syndrome is characterized by an ECG pattern of right bundle branch block with coved or occasionally saddleback ST-segment elevation in leads V1 through V3, the absence of demonstrable structural heart disease, and a propensity for ventricular tachycardia, ventricular fibrillation, and sudden cardiac death. The Brugada syndrome is a hereditary cardiac disease associated with a gene mutation affecting cardiac sodium channel characteristics. This mutation leads to the ECG pattern described above and carries a high risk of sudden cardiac death in patients despite a structural normal heart [7]. Occasionally the characteristic Brugada pattern is transient and may be exposed by a sodium channel blocker, such as flecainide, or procainamide in patients with “latent” Brugada syndrome [5]. Patients with ventricular dysrhythmias and heart block resulting from cocaine use should receive standard therapy, including the treatment of ischemia (if present), the correction of metabolic disturbances (e.g., electrolyte abnormalities, hypoxemia, or acid-base disturbances), the administration of appropriate antiarrhythmic agents, and temporary pacing, if indicated [1]. Cocaine has no known antidote. Benzodiazepines such as lorazepam remain the cornerstone of therapy and are considered first line treatment of cocaine-related myocardial injury. Malignant hypertension may be treated with direct vasodilators such as sodium nitroprusside. Beta-blockers should be avoided in suspected cocaine toxicity. Nonselective beta blocking agents, such as propranolol, may exacerbate cocaine-induced vasoconstriction of the coronary arteries [1, 3]. Several reports have described the successful treatment of cocaine-induced wide complex tachycardia with the administration of sodium bicarbonate. The increase in extracellular pH with sodium bicarbonate may have reversed impaired conduction by increasing the unionized fraction of cocaine, resulting in a more rapid dissociation of the drug from the sodium channels and a faster recovery from the sodium channel blockade [8].

## 4. Conclusion

Significant cocaine toxicity presents with neurologic and cardiovascular symptoms. Sudden death can occur and may be associated with respiratory paralysis secondary to the cocaine-related brainstem effects and subsequent asystole. Care is supportive, and benzodiazepines are first line agents to blunt sympathetic tone and treat seizures. Nitroglycerine should be added if cocaine-related myocardial ischemia is present. Cocaine is a sodium channel blocker and can unmask latent Brugada syndrome in patients with the susceptible gene. The natural history and significance of the transient Brugada pattern associated with cocaine abuse are unknown.
